# Hedonic response sensitivity to variations in the evaluation task and culinary preparation in a natural consumption context

**DOI:** 10.3389/fnut.2022.1008577

**Published:** 2022-10-28

**Authors:** Adriana Galiñanes Plaza, Laure Saulais, Julien Delarue

**Affiliations:** ^1^UMR SayFood, AgroParisTech, INRAE, Université Paris-Saclay, Palaiseau, France; ^2^Department of Agricultural Economics and Consumer Science, Laval University, Québec, QC, Canada; ^3^Institute Paul Bocuse, Ecully, France; ^4^Department of Food Science and Technology, University of California, Davis, Davis, CA, United States

**Keywords:** hedonic response, consumer evaluation, food testing, synthetic task, analytical task, multicomponent food, culinary preparation

## Abstract

Hedonic measurements in the frame of consumer tests of foods are prone to many different biases and the validity of test designs has been subject to much research with special emphasis on the role of context. While bringing elements of natural consumption context to the testing conditions is generally seen as an improvement, other aspects of the test design such as the task format have received little attention. In particular, the influence of analytical questions on hedonic responses has been studied in standardized contexts only. This study aimed to assess whether synthetic and analytical evaluation tasks result in different hedonic responses when the test is conducted in a natural consumption context. Bread and pizzas with different degrees of culinary preparation (homemade, readymade, and a combination of the two) were tested on three separate days in a university cafeteria. Overall liking scores of the bread and the three different pizzas were obtained either with a synthetic (hedonic question only) or with an analytical task (hedonic question plus intensity attributes). Care was taken to avoid any other changes to normal eating conditions, notably by recruiting on the spot only those customers who had spontaneously chosen pizza as part of their lunch. Liking scores of the homemade pizza were lower with the analytical task while the scores of the other two pizzas did not change significantly. Moreover, different rankings of the pizzas were obtained when the data were analyzed separately for each evaluation task format. The synthetic evaluation task would have led to the conclusion that the homemade pizza was the best liked and the readymade being the least liked, while the analytical evaluation task would have led to the conclusion that the “mixed” pizza would be liked significantly more than the other two. The effect of the task format (i.e., lower scores with the analytical task) was more pronounced when participants reported they had spent more time in the queue. These results strengthen the view that the task is part of the evaluation context and must be carefully considered when one wishes to design ecologically valid consumer tests.

## Introduction

Consumers' hedonic responses to foods and to other goods are commonly measured with rating scales in fields as diverse as sensory and consumer science, nutrition, and marketing. However, the context in which a study is conducted has been shown to potentially affect its outcome (1–4). Factors like physical location, social facilitation or availability of food options are suggested to explain why context may lead to different results (5).

In addition to these factors, test procedures and evaluation tasks may also contribute to differences in the outcome of hedonic tests. This type of context effects is referred to as *framing effects*, defined as the fact that the response to a question is linked to the way it is formulated (6). Framing effects have been attributed to the duality of cognitive processes that lead to judgment formation (7): an individual will rely either only on intuition (or, in the words of Kahneman: *system 1*) or on both intuition and reasoning (*system 2*) depending on the way the task involved in that judgment is framed.

Regarding food-related judgments, Köster (8, 9)suggested that differences in the way the evaluation task is formulated could induce varying levels of cognitive access to the attributes of the evaluated product. Indeed, the simple act of asking “Do you like this product?” or “Rate the flavor intensity of this product” is likely to induce reasoning, leading test participants to adopt a more analytical mindset than in a regular and more natural consumption situation where consumers may not explicitly ask themselves such questions (8–10). This issue has sparked interest, and several studies have investigated task-related variations in hedonic responses and found differences depending on the number of questions (11), the order in which they are asked (12), or the way they are formulated (13). In other studies, however, the question format did not appear to alter hedonic responses (14–16).

Common tasks for hedonic evaluation procedures typically require consumers either to make global judgments (*synthetic* evaluation task) or to rate successively several sensory attributes in addition to the overall liking score (*analytical* evaluation task). The choice of one task rather than another may impact the judgment-making processes involved in the hedonic evaluation. For example, Prescott et al. (17) compared the hedonic responses obtained either with synthetic or analytical evaluation of a tea drink. They found that mean liking scores were significantly higher when using a synthetic evaluation task than when using an analytical evaluation task. The authors argued that asking several questions to consumers such as rating sensory attributes may induce an analytical mind-set that undermines consumers' ability to engage the synthetic attentional approach that underlies hedonic responding. Consumers are thereby forced to resort to reasoning and to focus their attention on specific product characteristics, hence modulating their hedonic responses, while synthetic tasks may principally trigger intuitive judgment.

It is worth noting that Prescott et al. (17) results were observed in controlled testing conditions, where consumers' attention may be more focused on the task. It is not known whether such effects would be similar in natural consumption situations, where the attentional focus on both the task and on products' characteristics may differ due to the multitude of sensory stimuli surrounded the individual and the conditions involved [high cognitive load (18), level of hunger (19) or time constraints (20)]. In fact, most studies on the effect of the task format on hedonic responses were conducted in standardized environments, such as sensory labs or central testing rooms.

Yet, a recent study conducted by Zandstra et al. (21) investigated those effects on liking and Just-About-Right scores for four tomato soups in controlled, immersive and natural consumption situations. It showed no differences between the three contexts. However, despite efforts to make the physical context natural, participants in the dining out situation could not choose their food and sat with other participants that they did not know. Thus, the evaluation task could still be deemed somewhat artificial. In addition to this, the study was conducted according to a within-subject design (meaning that participants repeated the task in the three contexts), which may have also entailed the ecological validity of the natural consumption setting. Therefore, from that study, it seems difficult to draw conclusions on the role of evaluation tasks on hedonic responses in natural consumption situations.

As an attempt to shed light on this issue, we conducted a field study involving either a synthetic or an analytical evaluation task in a university restaurant in France. In order to keep the eating situation as natural as possible, we designed the study to survey regular customers without pre-recruitment. They paid for their meal; they were left completely free to choose their food, and to interact with others as they normally do when dining in the restaurant.

Following a protocol similar to that of Prescott et al. (17), we examined consumers' hedonic responses for food products using either a synthetic (overall liking) or an analytical questionnaire (overall liking plus attributes intensity scale). Secondly, previous studies having shown that context effects could depend on the product category (22), we studied the potential effect of the evaluation task on two product types (pizza and bread). These two products are normally served in that restaurant and are thus expected to be very familiar to customers. In order to assess how the evaluation task would possibly affect the differentiation between variants of the same product, we chose to test three variants of the pizza that is normally served and that was thus considered as a reference product. These variants underwent different culinary preparation and were served on separate days to simplify our logistics and avoid any confusion. However, knowing that contextual fluctuations are inevitable when conducting a field study, both versions of the questionnaire were tested each day according to a between-subject design. Besides, we monitored how consumers perceived their overall lunch experience to account for potential differences from 1 day to another. By contrast, to serve as a control point, we tested only one type of bread throughout the study.

Following Prescott et al. (17) findings, we hypothesized that the synthetic task would lead to higher hedonic scores than the analytical task. Furthermore, other studies having shown that more natural evaluation conditions could lead to higher hedonic discrimination between evaluated products (22, 23), we expected the synthetic evaluation task–deemed more natural–to potentially lead to larger differences in hedonic scores between the pizza variants.

## Materials and methods

### Participants

The research was conducted at the staff and student cafeteria of the Ecole Centrale of Lyon, France (a higher education institute with no major related to food science nor to consumer science). Four hundred and seventy three participants (24 ± 8 years old, 74% men) took part in the study. Participants were randomly assigned to different type of task questionnaire at their lunchtime. Participants were informed that their responses would be confidential, and voluntarily agreed to take part.

### Products

Two different products were evaluated: *Margherita* pizza and bread. *Margherita* pizza was selected because it is a standard dish usually well appreciated by the cafeteria customers. It is a multicomponent food that can undergo multiple modifications in terms of culinary preparation without altering its visual appearance. Moreover, the food service company running the cafeteria was also interested in their customers' opinion on pizzas in the view of improving their offer.

Three versions of pizzas, with varying degrees of culinary preparation, were served, respectively, on 3 separate days, 1 week apart, to avoid any confusion in the preparation and potential comparison bias. The *Margherita* pizza normally served at the university restaurant is made with ready-made dough, while the tomato sauce and toppings are prepared by the chef. It is thus referred to as the “mixed” pizza. The two other variants were either entirely prepared by the chef (and referred to as “homemade”), or entirely readymade. These changes to the culinary preparation were not communicated to the customers and the denominations (homemade, readymade, and mixed) are only used here for clarity. Table 1 summarizes the differences between the three versions of pizza.

**Table 1:** Description of the main differences among the three versions of pizza.

**Versions of pizza**	**Homemade**	**Mixed**	**Readymade**
Dough	Homemade (Prepared by the chef)	Readymade	Readymade
Tomato sauce	Homemade (Prepared by the chef)	Homemade (Prepared by the chef)	Readymade

Individual pizzas were of 300 ± 5 g (individual portion size). Each type of pizza was prepared and served in different days but following the same procedure. The homemade dough and tomato sauce were prepared a day before the service. From the homemade dough (flour, yeast, water, salt), balls of 160 g were cut to follow the same size of the readymade dough (*Mademoiselle Desserts St Renan, France*) and they were kept at 4°C in the fridge. For the tomato sauce, ingredients were mixed the day before (tomato, oregano, basil, pepper, olive oil) and they were also kept at storage at 4°C. The day of the study, all preparations started at 6.30 am. The oven was turned on at 350°C and set at speed of 2.5. Both types of dough (a homemade dough for the homemade pizza and a readymade dough for the mixed pizza) were kneaded by using a pizza dough “paver” and then placed on dishes where the tomato sauce, cheese and olives were added. The readymade pizza (*Marie surgelés*, France) followed the same last step of the protocol where the cheese and olives were added. The pizzas were cooked in the oven and stored in a refrigerator (4°C) until the cafeteria was opened. Once the service started (11.30 am), the pizzas were re-heated in the oven at 350°C and at speed 2 on demand.

Bread is a popular and familiar staple food which is served every day at the cafeteria and consumed by a majority of customers. Contrary to the pizza, the type, recipe, and quality of bread was kept constant all along the study. It was served in 30 g individual portions (“mini-baguettes”). It was thus selected to serve as a reference product for evaluation across study days.

Pizza and bread were available as part of the menu during the 3 days of study. However, the bread was only evaluated during the first 2 days.

### Procedure

**Figure 1:**
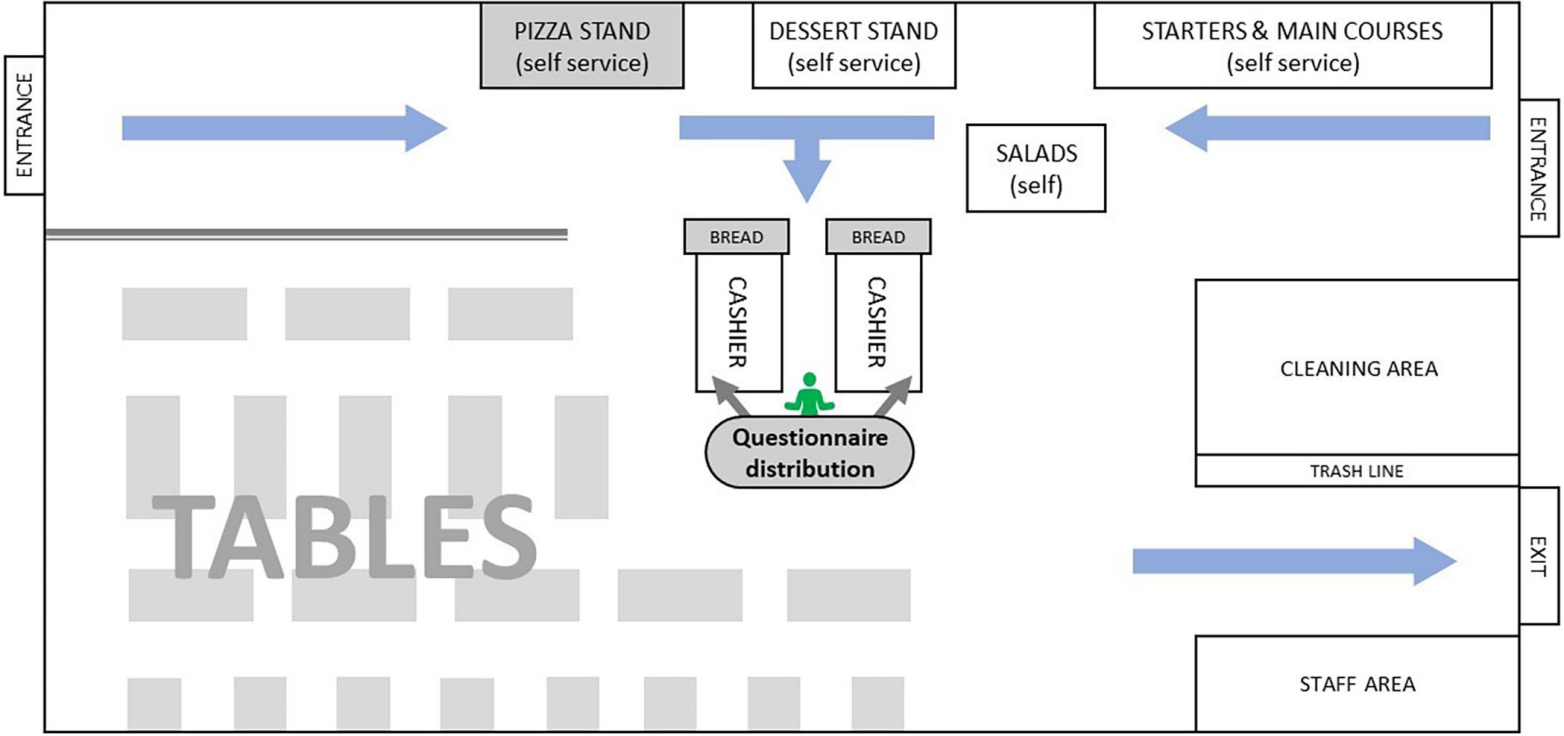
Plan of the staff and student cafeteria of the Ecole Central of Lyon, France.

Evaluations took place at the staff and student cafeteria of the Ecole Central of Lyon, France. Each evaluation was performed with a week apart and both versions of the questionnaire (synthetic or analytic) were handed out each testing day in a counterbalanced number. No information was given about the different versions of the pizza nor about the products concerned by the study and the cafeteria operated as usual without any change introduced. Participants arrived for lunch at the cafeteria from 11:30 to 14:00. Customers create their own fixed-price meal by choosing among three or four starters, four main dishes (pizza being one of them) and several desserts. Food items are presented on separate stands where customers help themselves (Figure 1). Once at the checkout counter, we spotted participants who had added to their trays the products that we were interested in, and we asked them whether they wanted to participate in the study, and if they could fill out a questionnaire. They were randomly given either a synthetic or an analytical version of the questionnaire. We told them to fill it while eating and to return it before leaving the cafeteria. Table 2 shows the design of the experiment regarding the tested products and their respective culinary modification and the evaluation task.

**Table 2:** Experimental design.

	**Week 1**		**Week 2**		**Week 3**	
	**Homemade**		**Mixed**		**Readymade**	
**Pizza**	**Synthetic task**	**Analytical task**	**Synthetic task**	**Analytical task**	**Synthetic task**	**Analytical task**
	***n* = 39**	***n* = 48**	***n* = 43**	***n* = 43**	***n* = 55**	***n* = 45**
	**Standard and unchanged recipe**				**No evaluation**	
**Bread**	Synthetic task	Analytical task	Synthetic task	Analytical task		
	*n =* 45	*n =* 50	*n =* 50	*n =* 55		

Following the protocol of Prescott et al. (17), we first asked participants about their liking on a 11-point hedonic scale with end-point labels (0 = dislike very much; 10 = like very much). This type of scale is more common to French consumers than the 9-point hedonic scale. For the analytical group, we also asked to evaluate a series of attributes related to the pizza or bread on a 11-point category scale with end-point labels (0 = very weak; 10 = very strong). The rated attributes were:

- Pizza: tomato flavor, saltiness, fattiness, cheese flavor, soft texture;
- Bread: saltiness, yeast flavor, soft crumb texture, crispiness of the crust, crunchy dough.

In addition to this, and on a separate page, the questionnaire included a short satisfaction survey, with two questions related to the main course [overall satisfaction; quality of the food (value-for-money)], and questions about participant's overall experience in the restaurant that day (time spent in the queue, ambiance, hunger before lunch, ate alone or with friends).

### Data analysis

Liking data were analyzed using a Student's independent *t* test for bread and using a two-way ANOVA with interaction for pizzas, where the type of culinary preparation and the type of task were included as main effects. When the ANOVA showed a significant effect (*p* < 0.05), a *post-hoc* Tukey HSD test was applied. In the case of bread, the effect of the evaluation task on overall liking was tested using an independent sample Student's *t*-test.

Data from the second part of the questionnaire (satisfaction survey) were analyzed using one-way ANOVA to check for potential differences between testing days. Special attention was paid to the possible effect of perceived time spent queueing on satisfaction and liking using simple linear regressions. Thereupon, an analysis of covariance (ANCOVA) with second order interaction was also performed to account for the effect of queueing (as a quantitative covariable) and of culinary preparation and evaluation task (as qualitative variables) on the liking scores. The resulting model was used to estimate corrected mean liking scores (LS Means) for each product in each condition.

All analyses were performed using XLSTAT 2022.2 (Addinsoft, statistical and data analysis solution. Paris, France).

#### Nota bene

We selected different participants each week. However, as the study was conducted in a natural consumption context, we cannot exclude that some participants took part of the study twice (e.g., on week 1 and 2). Should this have occurred, it would had been marginal. We thus treated the data from each day as independent groups.

## Results

### Pizza sensory description

Owing to our design, half of the participants rated their perception of the food for five sensory attributes. Data show that the three pizza variants clearly differed on the flavor of the tomato sauce, on the cheese flavor and on the texture of the crust (Table 3). The readymade pizza had a more intense tomato flavor and cheese flavor as well as a softer texture. There were no significant differences in terms of fattiness and saltiness.

**Table 3:** Analyses of variance of the sensory attributes of the different types of pizza preparations.

	**Tomato flavor**	**Salty flavor**	**Fatty**	**Cheese flavor**	**Soft texture**
*F*_(2, 134)_	8.51	1.64	1.99	6.65	4.89
*p*-value	< 0.001	0.197	0.141	0.002	0.009
Homemade	5.96 a	5.51 a	6.39 a	6.02 b	5.98 b
Mix	4.53 b	5.56 a	6.28 a	6.42 b	6.3 ab
Readymade	6.31 a	4.87 a	7.04 a	7.40 a	7.13 a

Letter indices indicate Tukey post-hoc groupings at p < 0.05 for each attribute.

### Overall liking

**Figure 2:**
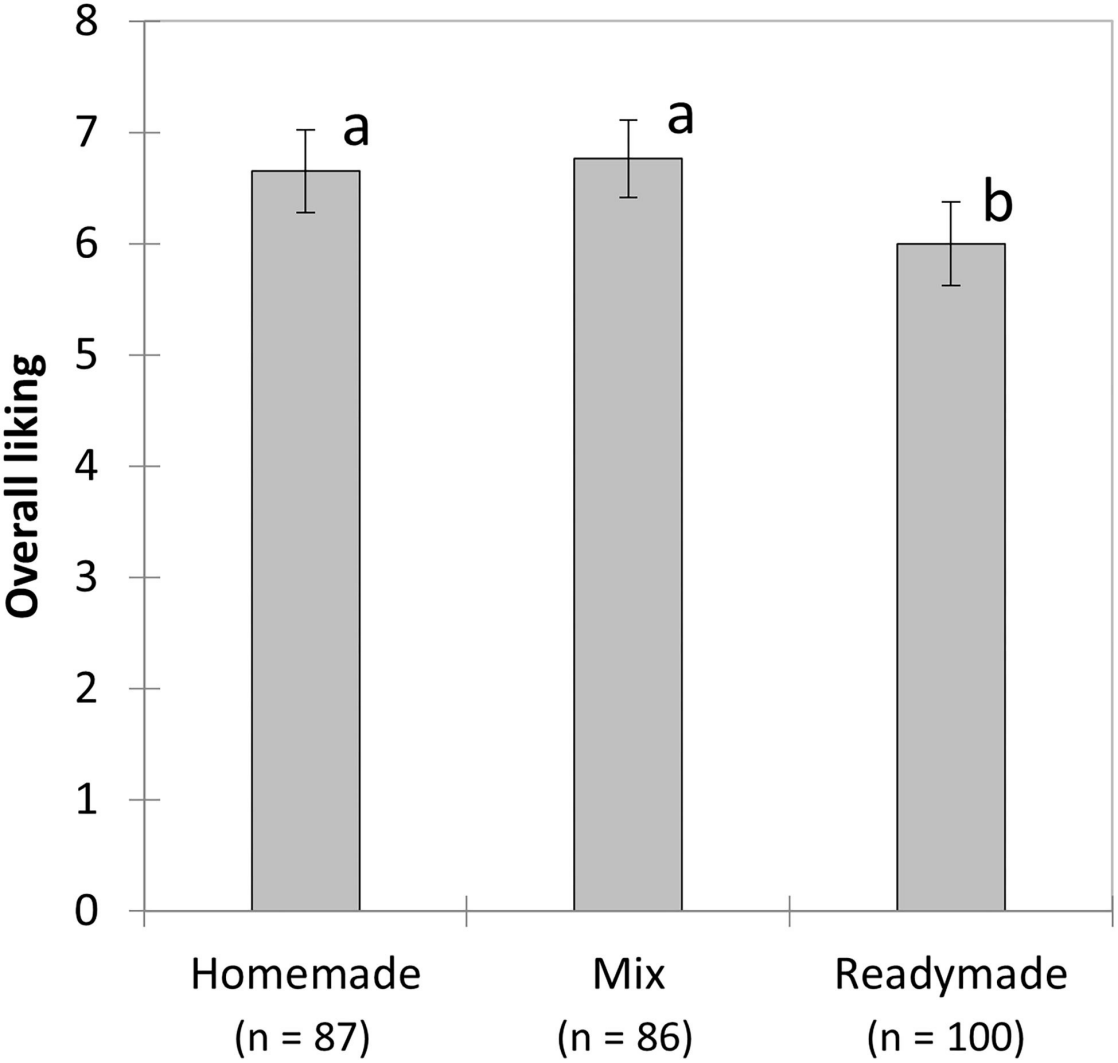
Mean scores (and SEM) for overall liking of the three pizza preparations. a and b indicate Tukey *post-hoc* groupings at *p* < 0.05.

Regardless of the evaluation task, pizzas were overall well liked with a mean score of 6.45 (±1.81), whereas bread was not so much appreciated [mean liking score: 4.51 (±1.90)]. On average, the pizza variants were differently liked (*F*_(2, 267)_ = 5.32, *p* = 0.005), with the homemade pizza and the mixed pizza receiving higher scores than the readymade pizza (Figure 2). The readymade pizza was less liked, possibly as a result of its softer texture, but its more intense cheese and tomato flavor could also have contributed to this outcome. However, analysis of the exit questionnaire revealed that time spent in the queue was perceived to be longer on the day the readymade pizza was served (*F*_(2, 267)_ = 10.42, *p* < 0.0001). On average, this seems to have reflected in overall satisfaction (*F*_(1, 267)_ = 6.36, *p* = 0.012, *R*^2^ = 0.02) and liking (*F*_(1, 267)_ = 6.94, *p* = 0.009, *R*^2^ = 0.02) even if interindividual differences were important, as indicated by the low coefficients of determination.

### Influence of the task format

**Figure 3:**
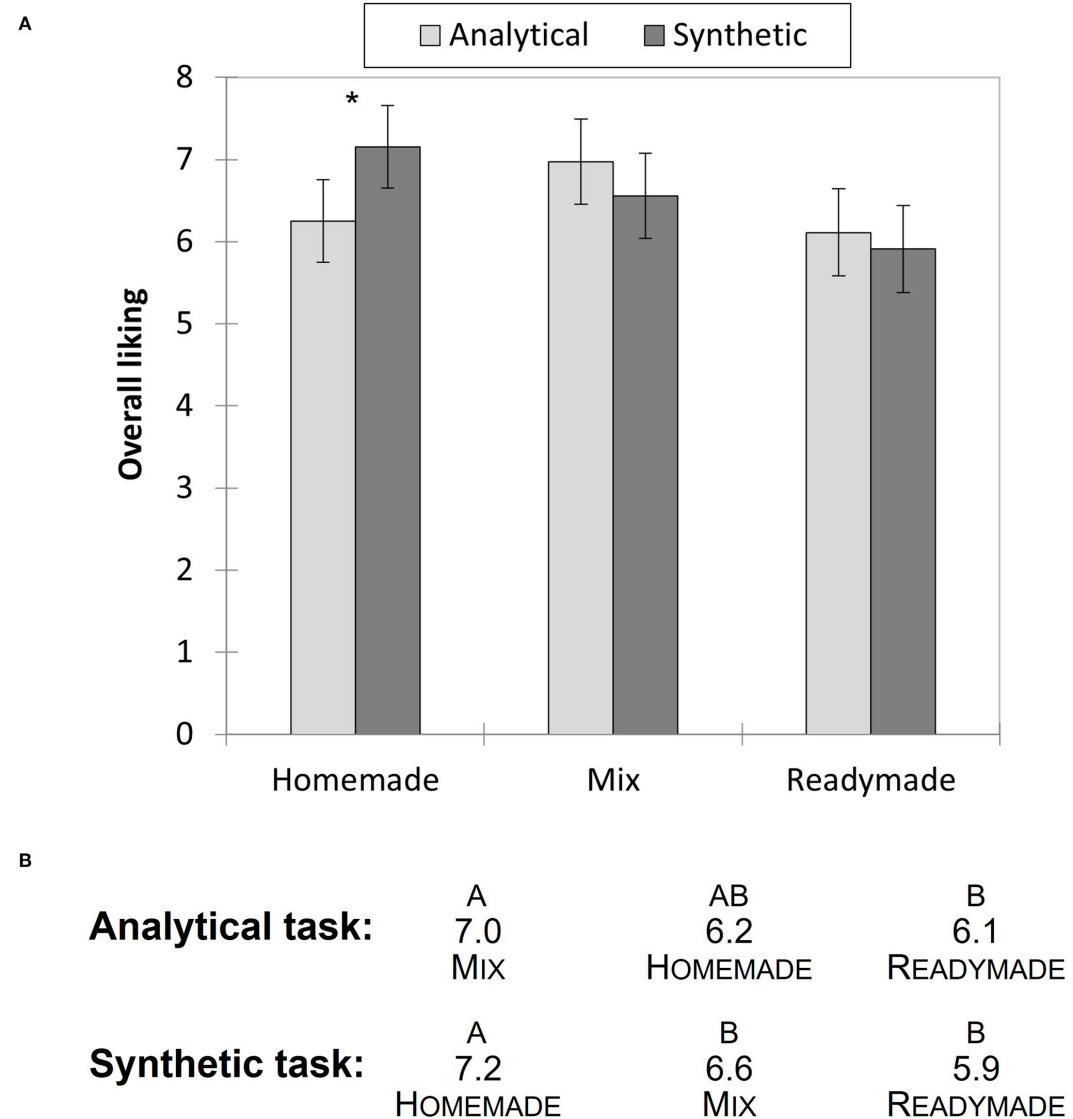
**(A)** Mean scores and standard errors for overall liking of the three pizza preparations for each task format, * indicates a significant difference at *p* < 0.05. **(B)** Rank order of the different pizza versions for the most liked to least liked according to each evaluation task. Letters above products denote significant differences (*p* < 0.05) found between each culinary preparation using *post-hoc* LSD test.

Overall, the task format did not influence the average liking score for the bread (*t*_(176)_ =1.97, *p* = 0.114), nor for the pizzas (*F*_(1, 267)_ = 0.19, *p* = 0.66). However, there was a significant interaction between the pizza preparation and the task format (*F*_(2, 267)_ = 3.51, *p* = 0.031), indicating that the pizza variants were scored differently depending on the questionnaire used (Figure 3A). In particular, the average liking score for the homemade version was significantly lower when participants performed the analytical evaluation task (*t*_(85)_ =2.86, *p* = 0.005).

What is more, different rankings of the pizzas were obtained when the data were analyzed separately for each evaluation task format (Figure 3B). With the synthetic task, the homemade was the best liked pizza, followed by the mixed (although not statistically different) and the readymade being the least liked. In contrast, the “mixed” pizza was significantly better liked than the other two when the analytical task was used. These rankings do not reflect individual preferences since the test was conducted in a pure monadic way. However, if a food service company had tested their products in such conditions, they would have reached different conclusions depending on the questionnaire format, and possibly different decision on which preparation or which recipe to select. Note that the synthetic evaluation task was slightly more discriminant than the analytic task, although effect sizes were very similar (Synthetic task: *F*_(2, 134)_ = 5.29, *p* = 0.006, η^2^ = 0.07; Analytic task: *F*_(2, 135)_ = 3.34, *p* = 0.039, η^2^ = 0.05).

In order to account for the effect of queueing on liking, we performed an ANCOVA (Table 4), which revealed that, in fact, the task format had a significant effect on the liking scores for the pizza. According to this model, the synthetic task indeed led to slightly higher adjusted mean scores (LS mean _synthetic_ = 6.55 ± 0.15 SE) than the analytical task (LS mean _analytic_ = 6.45 ± 0.17 SE). This analysis confirms the significant interaction between the task format and the pizza preparation that was previously observed. The adjusted mean score for the homemade pizza is now clearly higher when evaluated with the synthetic task (LS mean _synthetic_ = 6.97 ± 0.29 SE) than with the analytic task (LS mean _analytic_ = 5.82 ± 0.28 SE).

**Table 4:** Detailed ANCOVA model for the analysis of pizza liking scores (*F*_(9, 259)_ = 3.77, *p* < 0.001).

**Source**	**df**	**Sum of squares**	**Mean squares**	***F***	***p*-value**
**Queueing**	**1**	**27.34**	**27.34**	**9.16**	**0.003**
**Task format**	**1**	**18.28**	**18.28**	**6.12**	**0.014**
Preparation method	2	5.84	2.92	0.98	0.378
Queueing*Task format	**1**	**20.26**	**20.26**	**6.78**	**0.010**
Queueing*Preparation method	2	9.67	4.83	1.62	0.200
Task format*Preparation method	**2**	**32.18**	**16.09**	**5.39**	**0.005**

Significant sources of variation are highlighted in bold.

Interestingly, we identified a significant interaction between the queueing and the task format, indicating that the effect of the task format (i.e., lower scores with the analytical task) was more pronounced when participants spent more time in the queue (*t*_*slopes*_ = 2.605, *p* = 0.010).

## Discussion

The format of the evaluation task significantly impacted consumer hedonic responses for one of the tested products. The analytical task indeed resulted in lower hedonic scores than the synthetic task for the homemade pizza, hence echoing Prescott et al. (17) observation for iced tea. However, this effect did not affect all pizza preparations, and in parallel, bread, whose recipe did not change across the experimental campaign, received consistent scores with both types of tasks. Thus, contrary to our first hypothesis, we cannot conclude on a systematic effect of the evaluation task on the level of liking for all tested products.

The fact that the sensitivity to the task format apparently depends on the tested product could be highly consequential in a business context. For example, in foodservice, such test results would typically be used to evaluate liking for new products or new recipes and to decide which product to serve to customers, or to launch on the market. Here, in the case of pizzas, the two tasks would have been conducive to a different outcome in terms of order of preference and thus different decisions been made about which variant to offer. The synthetic evaluation task led to the conclusion that the homemade pizza was the best liked and the readymade the least liked, while the analytical evaluation task (which is more often used in satisfaction surveys in cafeterias) led to the conclusion that the “mixed” pizza was liked significantly better than the other two.

It should be noted that the mixed pizza was the regular product usually served in this cafeteria. Familiarity may thus have contributed to the observed differences in the relative impacts of analytical and synthetic tasks on evaluations outcomes (24–26). Previous research in behavioral economics suggests the existence of a link between the level of expertise, or familiarity, with a task and the use of judgment heuristics. For instance, in a market experiment, participants that were more familiar with the experimental task (an auction mechanism) were less subjected to the influences of the task context, in particular to endowment effects (27). No work has, to our knowledge, examined this relationship between the level of familiarity and the reliance on contextual cues within the context of food products evaluation tasks. However, it may be hypothesized that for more familiar products, evaluators would rely less on task-related cues, such as the criteria provided by analytical tasks. In our experiment, the mixed pizza was regularly served in this cafeteria and arguably the most familiar to customers. For this product, the liking scores were not significantly different between analytical and synthetic tasks, suggesting a low influence of the additional contextual cues (specific attributes) provided in the analytical task. A similar behavior was observed in the case of the readymade pizza, which is a familiar product in the population studied (students), and for bread, which is also a familiar and frequently consumed product. Conversely, the least familiar homemade pizza scored higher with the synthetic task than when participants' attention was focused on specific sensory attributes.

Interestingly, the task format did not influence the liking for bread, which received much lower liking scores overall than pizzas. The reasons are unclear why some products were affected while others were not. However, the result for bread is consistent with previous observations that liking scores are more sensitive to the task format for highly liked products than for disliked products (12, 13). This might also explain why, in our study, the task format did not affect the scores of the less liked pizzas. It can also be stressed that, contrary to bread, pizza is a main course and is a multicomponent food composed of multiple easily distinguishable subparts such as toppings (meat, cheese, etc.), tomato sauce, and crust, which could have been evaluated separately. The analytical task, which focuses on a selected set of sensory attributes, may have modulated the participants' overall liking scores by directing their attention on distinctive subparts (28). It would be interesting to test this hypothesis with other types of “homogeneous” (e.g., fruit juices, yogurts, cakes, etc.) and multicomponent (e.g., fruit bowls, salads, sushi, sandwiches, etc.) foods.

We can only speculate about which factors may have contributed to the observed differences in the relative impacts of analytical and synthetic tasks on evaluations outcomes. However, our results are in line with behavioral research that stresses the importance of contextual cues and reference points on judgment and decision-making, underlining that some judgments are led by intuition and rely more heavily on contextual cues, while others mobilize a more analytical and reflexive evaluation process (7, 29, 30).

In addition to the changes in the evaluation task induced by the use of different questionnaires, we measured the effect of variables that couldn't be controlled such as the perception of the time spent in the queue, the general ambiance, or whether participants ate alone or with friends / colleagues. As it happened, the time spent queuing was perceived to be significantly longer on the day the readymade pizza was served, which seemed to have negatively affected the liking scores for that pizza. Unfortunately, we did not collect data for bread on that day and cannot use this “control” product to back this hypothesis. However, this observation is consistent with previous studies that showed that queueing could influence liking and food choices in a cafeteria context (31, 32). Our model shows that when accounting for the perceived waiting time, the task format significantly affects liking scores for all pizzas, with lower liking scores when the analytical task was used. What is more striking, we found that the analytical task led to even lower liking scores when participants reported to having spent more time in the queue. This could be seen as a halo effect of the negative attitude induced by the waiting time. Should this be the case, it would suggest that longer and more analytical questionnaires would be more sensitive to such negative contextual events. This draws attention to the interaction of the task format and the evaluation context, and the potential associated biases. Rather, we would claim that the task is part of the evaluation context and must be carefully considered when one wishes to design ecologically valid consumer tests. Conversely, our results show that it would be hazardous to generalize conclusions on task effects drawn from tests conducted in one specific context, especially if this context (e.g., a sensory booth) remotely compares with real consumption situations.

Eventually, we would like to stress that this study was a field experiment, which involved a wide range of food options and possible selection biases as participants were recruited after they had selected their food and paid for their lunch. Although such an approach is seen to best represent the context in which consumers naturally behave and make decisions, the downside is the lack of control over some evaluation conditions (33). A crowdy day and longer queue is a typical example of such undesirable effects. Besides, we could only reach relatively small sample size in each condition, to be compared with the large number of participants overall (because we only recruited those consumers who spontaneously picked pizza for their meal among a much wider assortment). Despite these limitations, field experiments have high ecological validity (i.e., realistic representation of the studied stimuli in an natural environment). In this realistic environment, we find that the outcomes of satisfaction surveys for new recipes may be sensitive to the task design. Consistently with most studies on context, it was clear that many intrinsic and extrinsic variables could come into play (9). Accordingly, our results highlight the need to replicate this study, ideally with foods varying in the way they are eaten and in the type of expectations they convey.

## Data availability statement

The raw data supporting the conclusions of this article will be made available by the authors, without undue reservation.

## Ethics statement

Ethical review and approval was not required for the study on human participants in accordance with the local legislation and institutional requirements. The patients/participants provided their written informed consent to participate in this study.

## Author contributions

AG designed the study, collected data, analyzed and interpreted data, and drafted and revised the manuscript. LS designed the study, interpreted data, and revised the manuscript. JD designed the study, analyzed and interpreted data, and drafted and revised the manuscript. All authors contributed to the article and approved the submitted version.

## Funding

This work is part of a PhD project funded by the Société Scientifique d'Hygiène Alimentaire (SSHA), Paris, France.

## Conflict of interest

The authors declare that the research was conducted in the absence of any commercial or financial relationships that could be construed as a potential conflict of interest.

## Publisher's note

All claims expressed in this article are solely those of the authors and do not necessarily represent those of their affiliated organizations, or those of the publisher, the editors and the reviewers. Any product that may be evaluated in this article, or claim that may be made by its manufacturer, is not guaranteed or endorsed by the publisher.

## References

[1] Delarue J Boutrolle I. The effects of context on liking : implications for hedonic measurements in new product development. In: Consumer-Driven Innovation in Food and Personal Care Products, editors Sara R.J. Hal M (Cambridge: Woodhead Publishing) (2010). p. 175–218. 10.1533/9781845699970.2.175

[2] Galiñanes Plaza A Saulais L Delarue J. What really matters when dining out? Insights into the role of context from a qualitative study with French consumers. Int J Gastron Food Sci. (2022) 28:100537. 10.1016/j.ijgfs.2022.100537

[3] Jaeger SR Roigard CM Le Blond M Hedderley DI Giacalone D. Perceived situational appropriateness for foods and beverages: consumer segmentation and relationship with stated liking. Food Qual Prefer. (2019) 78:103701. 10.1016/j.foodqual.2019.05.001

[4] Meiselman HL. Context. The Effects of Environment on Product Design and Evaluation 1st Ed. Duxford: Woodhead Publishing (2019).

[5] Galiñanes Plaza A Delarue J Saulais L. The pursuit of ecological validity through contextual methodologies. Food Qual Prefer. (2019) 73:226–47. 10.1016/j.foodqual.2018.11.004

[6] Cartwright E. Behavioral Economics 3rd ed. London: Routledge (2018). 10.4324/9781315105079-1

[7] Kahneman D. Maps of bounded rationality: a perspective on intuitive judgement. In: Frangsmyr T, Editor. Nobel Prizes 2002: Nobel Prizes, Presentations, Biographies, and Lectures. Almqvist and Wiksell Int (2002). p. 449–89.17631876

[8] Köster EP. The psychology of food choice: some often encountered fallacies. Food Qual Prefer. (2003) 14:359–73. 10.1016/S0950-3293(03)00017-X

[9] Köster EP. Diversity in the determinants of food choice: a psychological perspective. Food Qual Prefer. (2009) 20:70–82. 10.1016/j.foodqual.2007.11.002

[10] Boutrolle I Delarue J Köster E Aranz D Danzart M. Use of a test of perceived authenticity to trigger affective responses when testing food. Food Qual Prefer. (2009) 20:418–26. 10.1016/j.foodqual.2009.03.004

[11] Spinelli S Masi C Dinnella C Zoboli GP Monteleone E. How does it make you feel? a new approach to measuring emotions in food product experience. Food Qual Prefer. (2014) 37:109–22. 10.1016/j.foodqual.2013.11.009

[12] Earthy PJ MacFie HJH Hedderley D. Effect of question order on sensory perception. J Sens Stud. (1996) 12:215–37. 10.1111/j.1745-459X.1997.tb00064.x

[13] Popper R Rosenstock W Schraidt M Kroll BJ. The effect of attribute questions on overall liking ratings. Food Qual Prefer. (2004) 15:853–8. 10.1016/j.foodqual.2003.12.004

[14] Gacula MJR Mohan P Faller J Pollack L Moskowitz HR. Questionnaire practice: What happens when the jar scale is placed between two “overall” acceptance scales? J Sens Stud. (2008) 23:136–47. 10.1111/j.1745-459X.2007.00147.x

[15] Jaeger SR Giacalone D Roigard CM Pineau B Vidal L Giménez A. Investigation of bias of hedonic scores when co-eliciting product attribute information using CATA questions. Food Qual Prefer. (2013) 30:242–9. 10.1016/j.foodqual.2013.06.001

[16] Jaeger SR Hunter DC Kam K Beresford MK Jin D Paisley AG. The concurrent use of JAR and CATA questions in hedonic scaling is unlikely to cause hedonic bias, but may increase product discrimination. Food Qual Prefer. (2015) 44:70–4. 10.1016/j.foodqual.2015.04.001

[17] Prescott J Lee SM Kim KO. Analytic approaches to evaluation modify hedonic responses. Food Qual Prefer. (2011) 22:391–3. 10.1016/j.foodqual.2011.01.007

[18] Dai J Cone J Moher J. Perceptual salience influences food choices independently of health and taste preferences. Cogn Res Princ Implic. (2020) 5:2. 10.1186/s41235-019-0203-2 31900744 PMC6942074

[19] Gidlöf K Ares G Aschemann-Witzel J Otterbring T. Give us today our daily bread: The effect of hunger on consumers' visual attention towards bread and the role of time orientation. Food Qual Prefer. (2021) 88. 10.1016/j.foodqual.2020.104079

[20] Massey C Brémaud D Saulais L. Sandwich or long lunch? lack of time and attendance of food outlets by French workers. Int J Workplace Health Manag. (2021) 14:164–80. 10.1108/IJWHM-05-2020-0084

[21] Zandstra EH Kaneko D Dijksterhuis GB Vennik E De Wijk RA. Implementing immersive technologies in consumer testing: liking and just-about-right ratings in a laboratory, immersive simulated café and real café. Food Qual Prefer. (2020). 84:103934. 10.1016/j.foodqual.2020.103934

[22] Boutrolle I Delarue J Arranz D Rogeaux M Köster EP. Central location test vs. home use test: contrasting results depending on product type. Food Qual Prefer. (2007) 18:490–9. 10.1016/j.foodqual.2006.06.003

[23] Bangcuyo RG Smith KJ Zumach JL Pierce AM Guttman GA Simons CT. The use of immersive technologies to improve consumer testing: the role of ecological validity, context and engagement in evaluating coffee. Food Qual Prefer. (2015) 41:84–95. 10.1016/j.foodqual.2014.11.017

[24] Donadini G Fumi MD Porretta S. Influence of preparation method on the hedonic response of preschoolers to raw, boiled or oven-baked vegetables. LWT Food Sci Technol. (2012) 49:282–92. 10.1016/j.lwt.2012.07.019

[25] Donadini G Spigno G Porretta S. Preschooler liking of meal components: The impact of familiarity, neophobia, and sensory characteristics. J Sens Stud. (2021). 36:e12649. 10.1111/joss.12649

[26] Kim Y-K Jombart L Valentin D Kim K-O. Familiarity and liking playing a role on the perception of trained panelists: a cross-cultural study on teas. Food Res Int. (2015) 71:155–64. 10.1016/j.foodres.2015.03.022

[27] List JA. Does market experience eliminate market anomalies? Q J Econ. (2003) 118:41–71. 10.1162/00335530360535144

[28] Cardello AV. Hedonic scaling: assumptions, contexts and frames of reference. Curr Opin Food Sci. (2017) 15:14–21. 10.1016/j.cofs.2017.05.002

[29] Liu D Juanchich M Sirota M Orbell S. The intuitive use of contextual information in decisions made with verbal and numerical quantifiers. Q J Exp Psychol. (2020) 73:481–94. 10.1177/1747021820903439 31952448 PMC7502984

[30] Moran AJ Khandpur N Polacsek M Rimm EB. What factors influence ultra-processed food purchases and consumption in households with children? a comparison between participants and non-participants in the supplemental nutrition assistance program (SNAP). Appetite. (2019) 134:1–8. 10.1016/j.appet.2018.12.009 30550893

[31] Edwards JSA Hartwell HJ Price S. The effects of environment on product design and evaluation: meals in context, institutional foodservice. In: Meiselman HL, Editor. Context. The Effects of Environment on Product Design and Evaluation. Duxford: Woodhead Publishing (2019). p. 259–85. 10.1016/B978-0-12-814495-4.00013-1

[32] Ryan D Holmes M Ensaff H. Adolescents' dietary behaviour: the interplay between home and school food environments. Appetite. (2022) 175:106056. 10.1016/j.appet.2022.106056 35447162

[33] Meiselman HL. Methodology and theory in human eating research. Appetite. (1992) 19:49–55. 10.1016/0195-6663(92)90235-X 1416936

